# Spontaneous rectus sheath hematoma complicated by deep venous thrombosis: A case report

**DOI:** 10.1097/MD.0000000000040198

**Published:** 2024-10-25

**Authors:** Isaac Cheruiyot, Prabjot Sehmi, Vincent Kipkorir, Jeremiah Munguti, Julius Ogeng’o

**Affiliations:** a Department of Human Anatomy and Medical Physiology, Faculty of Health Sciences, University of Nairobi, Nairobi, Kenya.

**Keywords:** anticoagulation, hematoma, rectus sheath

## Abstract

**Rationale:**

Rectus sheath hematoma (RSH) is an unusual cause of acute abdominal pain. It is more common in elderly patients on anticoagulation. The diagnosis is often challenging, as it mimics other more common causes of acute abdomen. No standard treatment guidelines exist, presenting therapeutic dilemmas. Herein, we describe a case of spontaneous RSH complicated by deep venous thrombosis (DVT).

**Patient concerns:**

The patient was a 58-year-old female on follow-up for end-stage renal failure, admitted for management of uremic gastritis, fluid overload state, and bronchopneumonia. On the third day of admission, she developed worsening abdominal pains, associated with dizziness, headaches, hypotension, tachycardia, and desaturation. Abdominal examination revealed tender swelling, with localized guarding.

**Diagnoses:**

An urgent abdominal computed tomography scan demonstrated a large complex collection of approximately 12 cm × 10 cm in the left rectus sheath with intraperitoneal extension, consistent with RSH.

**Interventions:**

She underwent laparotomy with evacuation of 1.5 L of hematoma. Her postoperative recovery was complicated by the development of DVT on the sixth postoperative day. Due risk of rebleed, the inferior vena cava filter was favored over therapeutic anticoagulation. Her postoperative hospital stay was thereafter unremarkable.

**Outcomes and lessons:**

Although rare, RSH is a potential cause of acute abdomen, particularly among patients on anticoagulation, and can be life-threatening. A high index of suspicion is therefore important for early diagnosis. Clinicians should also appreciate the heightened risk of DVT in the immediate postoperative period despite mechanical anticoagulation.

## 1. Introduction

First described by Richardson in 1857,^[1]^ rectus sheath hematoma (RSH) is a rare clinical entity and an unusual cause of the acute abdomen.^[2,3]^ It is caused by rupture of inferior (IEA) or superior epigastric arteries, or bleeding from rectus muscle tears.^[3]^ Its diagnosis is often challenging, resulting in delays in the initiation of appropriate management, with resultant morbidity and mortality.^[3,4]^ Although mild forms of RSH may be treated conservatively, severe cases require emergent endovascular and/or surgical interventions.^[4,5]^ We herein describe a case of severe spontaneous RSH complicated by deep venous thrombosis (DVT).

## 2. Case description

A 58-year-old female patient on follow-up for systemic arterial hypertension and end-stage renal failure presented to the hospital for management of uremic gastritis, fluid overload state, and bronchopneumonia. A temporary hemodialysis (HD) catheter had been inserted in the right femoral vein in another facility a day before the presentation. She came in with a history of epigastric pains, nausea and vomiting, bilateral leg swelling, abdominal swelling, mild exertional dyspnea, facial puffiness, persistent productive cough, and hotness of the body. At admission, she was febrile (T-37.6°C), normotensive (BP 134/65 mm Hg) with normal pulse rate (83/min) and oxygen saturation (92% on room air). Physical examination revealed bilateral pitting edema of the leg, moderate pallor, bilateral coarse lung crepitations, epigastric tenderness (without guarding or rigidity), and shifting dullness of the abdomen. Initial lab work showed leucopenia (white cell count 2.3), moderate normocytic anemia (8.9 g/dL), elevated urea (18 mmol/L), elevated creatinine (423 µmol/L), and moderate hypervolemic hyponatremia (121 mmol/L). Chest X-ray showed features of bilateral consolidations and mild pleural effusion. She was started on intravenous antibiotics, biweekly HD, blood transfusion, prophylactic anticoagulation, and other supportive treatments, with significant improvements in her symptoms.

**Figure 1.**
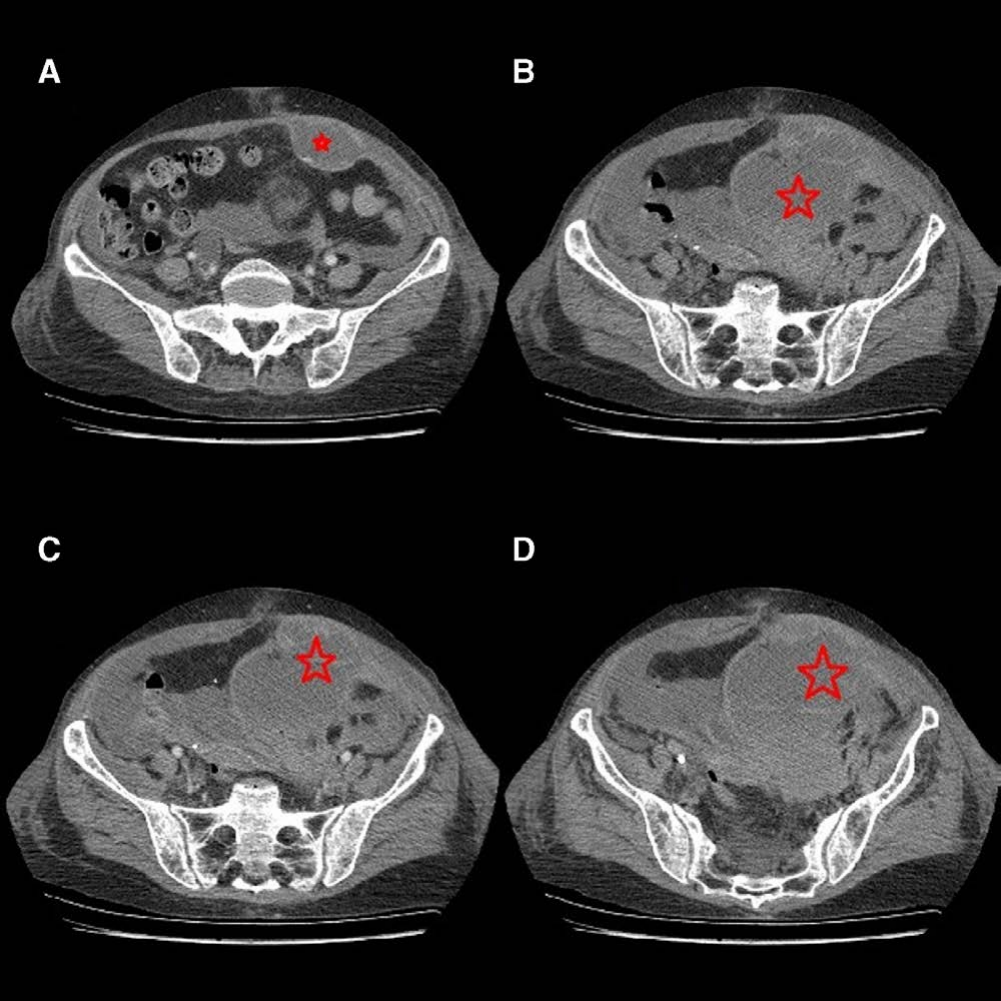
Cross-sectional CT scan of the abdomen showing the sheath hematoma. Star: Left rectus sheath hematoma. (A) No intraperitoneal extension. Note the intraperitoneal extension in B–D. CT = computed tomography.

On the third day of admission, she developed gradually worsening abdominal pains, associated with dizziness and headaches, hypotension (BP 80/54 mm Hg), tachycardia (PR 118), and desaturation (80% on room air). Initial resuscitation was done with the administration of oxygen (3l/minutes), insertion of a wide-bore cannula, drawing of samples (full blood count, grouping, and crossmatching), and judicial fluid resuscitation. Abdominal examination showed left upper/lower quadrant paramedian swelling, which was tender on palpation with localized guarding. The full hemogram showed hemoglobin had dropped to 4.8g/dL. An urgent abdominal computed tomography (CT) scan was done while receiving a transfusion of a pack of red blood cells. The abdominal imaging demonstrated a large complex collection of approximately 12 cm × 10 cm in the left rectus sheath with intraperitoneal extension, blood fluid level, and active flushing in the arterial phase (Fig. 1A–D). A diagnosis of left RSH was therefore made. The surgical team was brought on board. On account of the deranged coagulation profile (international normalized ratio 1.9, activated partial thromboplastin time >120 seconds), normalization of blood pressures with initial resuscitation with fluid/3 units of packed red cells, a decision was made to defer emergent laparotomy to allow for correction of abnormal coagulation parameters. She was started on protamine sulfate and vitamin K while continuing with transfusion and close monitoring of vitals in the high-dependency unit. The patient remained vitally stable for 36 hours, with repeat tests showing improved international normalized ratio (1.02) and activated partial thromboplastin time (40 seconds). However, abdominal pains are persistent despite attempts at optimization of parenteral analgesics. She therefore underwent laparotomy under general anesthesia. Approximately, 1.5 L of hematoma was evacuated from the left rectus sheath/pelvis. No active bleeding was encountered intraoperatively. Drains were placed to evacuate residual bleeds. Her immediate postoperative recovery was uneventful. She continued on biweekly HD and other supportive care. Due to the risk of rebleeding, prophylactic anticoagulation was held in favor of mechanical thromboprophylaxis.

On the 9th day of admission, she developed progressively worsening right lower limb pain and swelling, without any respiratory symptoms. Doppler ultrasonography demonstrated a large right common femoral vein thrombus. Due to the risk of rebleeding and pulmonary embolism, placement of an inferior vena cava (IVC) filter is favored over therapeutic pharmacological anticoagulation. The temporary right femoral vein HD catheter was also removed, and another one was placed in the internal jugular vein. With the abatement of admitting symptoms, absence of re-accumulation of hematoma, and no evidence of PE, the patient was allowed home. A review at follow-up showed no re-accumulation of the hematoma.

## 3. Discussion

RSH remains a relatively rare clinical condition. Development of DVT in the course of management of RSH is even more infrequent and, to the best of our knowledge remains hitherto undescribed. RSH accounts for approximately 1% of cases of acute abdomen.^[6]^ The true population incidence remains largely unknown. It is however more prevalent in those between 45 and 60 years, likely due to age-related muscle atrophy and higher rates of anticoagulant use within this age group.^[2,7]^

The source of the bleed is usually from either the superior epigastric artery or IEA.^[2,3]^ Similar to the current case, lower RSH from IEA tends to be larger, with intra- and/or retroperitoneal extension due to a deficient posterior rectus sheath wall below the arcuate line.^[2,7]^ Spontaneous RSH is by far the most common variety.^[4]^ Anticoagulation accounts for about 70% of such spontaneous cases,^[4,7,8]^ with muscle tears from causes such as chronic cough and pregnancy accounting for the rest.^[9]^ Anticoagulation (thromboprophylaxis and HD) appeared to be the risk factor for developing RSH in the current case. It is also plausible that the persistent cough could have contributed to the bleeding through muscle tears. The patient however had no history of abdominal trauma preceding the onset of the symptoms. Common traumatic causes include blunt abdominal trauma, paracentesis, and abdominal wall injections.^[6,10]^

Clinical presentation can be challenging, as it mimics other commoner causes of acute abdomen such as abdominal wall hernias and acute appendicitis.^[11]^ Typical symptoms include abdominal pain, mass, and overlying ecchymosis. The presence of nausea, vomiting, and constipation would suggest peritoneal and visceral irritation due to hematoma extension. Severe cases are usually accompanied by hemodynamic compromise, with episodes of tachycardia and hypotension, as seen in the current case. Only a small proportion of RSH (<15%) are incidental findings during abdominal imaging.^[3]^

Ultrasonography is often the initial imaging modality in most cases. This is due to its low costs, accessibility, and shorter turnaround time of results.^[4,12]^ It is however limited by its user-dependent nature, and may not be able to delineate RSH peritoneal extensions or concurrent intra-abdominal injuries.^[12]^ Cross-sectional abdominal CT scan has the highest sensitivity (100%) in the diagnosis of RSH.^[13]^ It can not only delineate the extent of the hematoma but also the presence of active bleeding and any concurrent intra-abdominal injuries, particularly in traumatic cases.^[13,14]^ Berná et al^[14]^ described a classification of RSH based on extent and laterality. Type 1 hematomas are intramuscular and unilateral, often fusiform/oval. Type 2 is dissected along fascial traversals but does not extend to the retropubic space. They can be unilateral or bilateral. Type 3 is usually large with retro- and or intraperitoneal extension.

Due to the rarity of the condition, there are no standard treatment guidelines for RSH. A high index of suspicion is, therefore, necessary for early recognition and institution of appropriate treatment. Treatment is usually dependent on the size/extent of the hematoma, hemodynamic stability, and coagulation profile of the patients.^[15]^ Conservative management is usually recommended in hemodynamically stable patients, with type 1 or 2 hematoma.^[15,16]^ This involves fluid resuscitation, transfusion or blood/blood products, reversal of anticoagulation, analgesia, and bed rest.^[15,16]^ This approach has been demonstrated to be effective in 50% to 80% of cases of RSH.^[15]^ Triggers of escalation of care include but are not limited to, hematoma expansion, peritoneal irritation, and hemodynamic instability.^[6]^ The endovascular approach is the first-line treatment for RSH not amenable to conservative management.^[17]^ The high costs, time-consuming nature, and unavailability are the major drawbacks to endovascular treatment of RSH.^[18,19]^ The clinical success rate of endovascular treatment is approximately 66%, with about a third of patients experiencing rebleeding, and are therefore candidates for surgical treatment.^[19]^ Surgical therapy allows for rapid control of bleeding via vascular ligation.^[6,18]^ It also allows evacuation of hematoma, which is useful in abatement of symptoms of peritoneal irritation. Surgical intervention can however be limited by abnormal coagulation profile due to a higher risk of excessive bleeding. In such cases, prompt initial resuscitation and reversal of anticoagulation play a vital role as a bridge to surgery. The timing of re-initiation of anticoagulation following treatment of RSH is also unclear. It is however informed by the risks of rebleeding and the benefits of anticoagulation.^[6]^ In the current case, anticoagulation was delayed in favor of mechanical anticoagulation. The patient in the current case however developed DVT, complicating postoperative management since the introduction of therapeutic doses of anticoagulation would significantly increase the risk of rebleeding. As such, insertion of an IVC filter was deemed to be the most appropriate option, as it would minimize the risk of pulmonary embolism, with no risk of re-accumulation of RSH.^[20]^ The IVC filters are effective in preventing PE in patients with contraindications to pharmacological thromboprophylaxis, with a reported failure rate of about 5% to 6%.^[20]^

## 4. Conclusion

Although rare, RSH is a potential cause of acute abdomen, particularly among patients on anticoagulation, and can be life-threatening. A high index of suspicion is important for early diagnosis. Subsequent management should be individualized, guided by hematoma size, peritoneal extents, coagulation profile, and hemodynamic stability. Clinicians should also appreciate the heightened risk of DVT in the immediate postoperative period despite mechanical anticoagulation.

## Author contributions

**Conceptualization:** Isaac Cheruiyot, Jeremiah Munguti.

**Data curation:** Isaac Cheruiyot.

**Investigation:** Isaac Cheruiyot.

**Methodology:** Isaac Cheruiyot.

**Writing – original draft:** Isaac Cheruiyot, Prabjot Sehmi, Vincent Kipkorir, Jeremiah Munguti, Julius Ogeng’o.

**Writing – review & editing:** Isaac Cheruiyot, Prabjot Sehmi, Vincent Kipkorir, Jeremiah Munguti, Julius Ogeng’o.
